# Beyond illness: Variation in haemosporidian load explains differences in vocal performance in a songbird

**DOI:** 10.1002/ece3.8455

**Published:** 2021-12-14

**Authors:** Salome Lopez‐Serna, Catalina Gonzalez‐Quevedo, Hector Fabio Rivera‐Gutierrez

**Affiliations:** ^1^ Instituto de Biología Facultad de Ciencias Exactas y Naturales Grupo de Ecología y Evolución de Vertebrados Universidad de Antioquia Medellín Colombia

**Keywords:** black‐striped sparrow, haemosporidian parasites, sexual selection, vocal performance

## Abstract

In animal communication, signals are expected to evolve to be honest, so that receivers avoid being manipulated by signalers. One way that signals can evolve to be honest is for them to be costly, with only high‐quality individuals being able to bear the costs of signal expression. It has been proposed that parasites can introduce costs that affect the expression of sexually selected traits, and there is evidence to support the role of parasitism in modulating animal behavior. If host infection status or intensity is found to relate to differences in signal expression, it may indicate a fitness cost that mediates honesty of signals. Birdsong is a good model for testing this, and physically challenging songs representing complex motor patterns provide a good example of sexually selected traits indicating individual condition. We performed a field study to evaluate the relationship between song performance and avian malaria infection in a common songbird. Previous work on this subject has almost always evaluated avian malaria in terms of binary infection status; however, parasitemia—infection intensity—is rarely assessed, even though differences in parasite load may have profound physiological consequences. We estimated parasitemia levels by using real‐time PCR. We found that birds with higher parasitemia displayed lower vocal performance, providing evidence that this song trait is an honest signal of parasitic load of haemosporidian parasites. To our knowledge, this study links parasite load and the expression of a sexually selected trait in a way that has not been addressed in the past. Studies using song performance traits and parasitemia offer an important perspective for understanding evolution of characters via sexual selection.

## Graphical abstract

We evaluated the role of avian malaria in evolution of sexually selected traits. Since differences in parasite load may have profound physiological consequences, we estimated parasitemia levels by using real‐time PCR. Higher parasitemia was associated with lower song performance, providing evidence that this song trait is an honest signal of parasitic load of haemosporidian parasites.

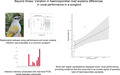

## 1 INTRODUCTION

It is well known that animals communicate actively, and during this information exchange, both signaler and receiver usually benefit from the information sent (Bradbury & Vehrencamp, 2011). While signalers may be under selection to manipulate receivers’ behavior to their own advantage (Stuart‐Fox, 2005), signals must, on average, be honest (Stuart‐Fox, 2005) because otherwise receivers would be selected to ignore them (Bradbury & Vehrencamp, 2011). Multiple factors are thought to maintain signal honesty: (i) signals must be costly, because production and maintenance require the expenditure of resources that could be allocated to other functions (Veiga, 1993); (ii) the cost and benefit obtained should vary between individuals (Searcy & Nowicki, 2005); and (iii) successful production of the signal should be correlated with the signaler's quality (Searcy & Nowicki, 2005; Wilson & Nussey, 2010).

Certain types of signal, such as birdsong, visual displays, body size, and ornaments, are commonly held to be sexually selected (Andersson & Iwasa, 1996). Sexual selection theory predicts that there is a relationship between these traits and reproductive success. In birds, song has been widely reported as serving as an indicator for male quality that should ultimately predict breeding and reproductive success (Soma & Garamszegi, 2011). Birdsong is a complex, multicomponent character (Gil & Gahr, 2002), and different features may be evaluated by the choosy sex in a species‐dependent manner. Indeed, numerous song traits have been found to correlate with reproductive success, including song structure (Nemeth et al., 2012; Woodgate et al., 2012), repertoire size (Robinson & Creanza, 2019), local song structure (O’Loghlen & Rothstein, 1995), “sexy syllables” (Vallet et al., 1998), syllable consistency (Botero et al., 2009), and vocal performance (Byers, 2007). A chooser might also assess multiple song traits, where different components of birdsong simultaneously carry multiple, redundant, or unreliable information (Rivera‐Gutierrez et al., 2010).

A case of honest signals was proposed by Hamilton and Zuk (1982), whereby secondary sexual characters in males can evolve to signal their parasite resistance to females. This may be true if parasites impose a fitness cost that moderates a trade‐off between character expression and fitness and by a negative relationship between parasite load and the expression of the trait (Garamszegi, 2005). Therefore, parasite presence, and parasite load, may increase character variability (Garamszegi, 2005; Laiolo et al., 2007) and covary with the expression of any directional sexual character. Phenotypic expression may then evolve to become coupled to signaler quality (De Lisle & Rowe, 2015).

In birds, blood parasites can have negative effects on important aspects of host life history and fitness components. Clutch size (Marzal et al., 2005), hatching success (Knowles et al., 2010; Sanz et al., 2001), fledgling success (Knowles et al., 2010; Merino et al., 2000; Pigeault et al., 2020), and offspring size (Szép & Møller, 2000) have all been found to be reduced by parasite infections, as have other aspects of reproductive behavior such as parental care (Ganser et al., 2020; Parejo‐Pulido et al., 2020). Ectoparasites, on the other hand, have been found to impose fitness costs on reproduction by affecting nestling body size and condition, and overall reproductive success in terms of fledgling success, fledgling survival, and thermogenic and metabolic capacities (Dufva & Allander, 1995; Fitze et al., 2004; Richner et al., 1993; Simon et al., 2004; Szép & Møller, 2000). Although it is clear that parasites affect life‐history traits, their effect on sexually selected displays, such as birdsong, remains poorly understood (Bischoff et al., 2009; Buchanan et al., 1999; Gilman et al., 2007; Müller et al., 2013; Redpath et al., 2000; Spencer et al., 2005).

In songbirds, parasite infection can affect song expression. Moreover, because birdsong has plastic components, it can also signal infection more immediately, thus having the potential to signal present health status (Buchanan & Catchpole, 1997; Laiolo et al., 2007; Müller et al., 2013). A way to signal individual quality via song performance is by having the ability to perform physically challenging songs that involve more complex motor patterns for their production (Ballentine et al., 2004; Podos, 1997, 2001). Trilled vocalizations (notes repeated in rapid succession) are mechanically and energetically challenging because they require rapid and precise vocal tract movements, with a trade‐off between note rate and the bandwidth in which they can be produced (Podos, 1997). On the other hand, trilled songs may be constrained by this trade‐off, and there is a performance limit, in which higher quality males may produce more challenging songs (Ballentine et al., 2004; Podos, 1997).

With the aim of understanding the role of parasite‐mediated expression of sexually selected signals, we performed a field study in which we evaluated song performance in trilled songs of a common songbird while determining avian malaria infection, a vector‐borne disease caused by haemosporidian parasites. Differences in haemosporidian load may have differential physiological consequences for hosts. Therefore, accurately measuring parasitemia could be important when evaluating the role of infection in influencing hosts’ behavior, physiology, or fitness (Schoenle et al., 2017). Studies on avian malaria have found that parasite presence is not necessarily directly proportional to infection intensity or parasitemia (Roth et al., 2021). Thus, studying the effects of parasitemia on life‐history traits of individual hosts is more insightful than analyzing infections in binary, presence/absence terms. We therefore estimated parasitemia levels by using real‐time polymerase chain reaction (PCR) to quantify parasite DNA and mapped those levels onto numerous song features that we hypothesized might be impacted by parasite presence and load.

## 2 METHODS

### 2.1 Study system

**FIGURE 1:**
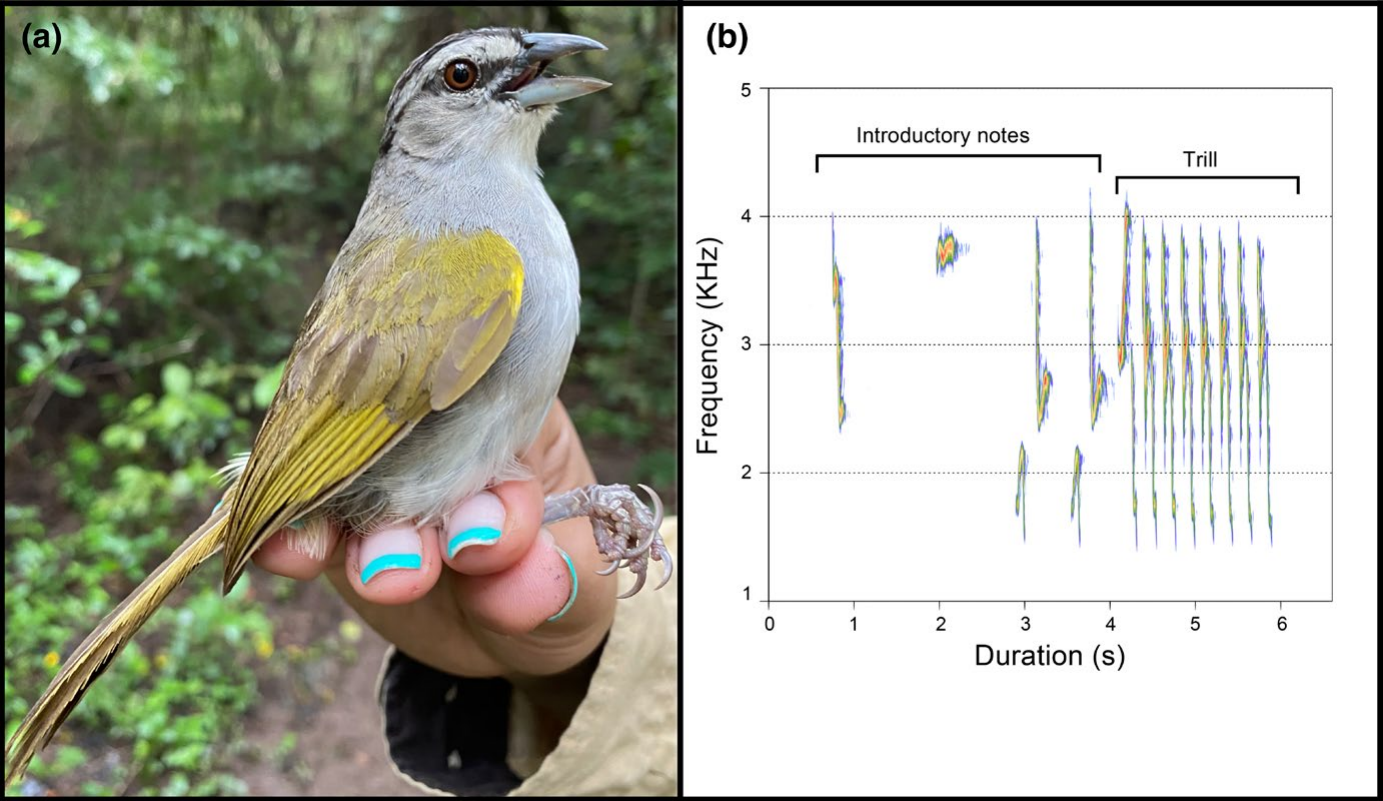
(a) Black‐striped sparrow (*Arremonops conirrostris*). Photo by Paula Pinzón, Ecology and Evolution Research group, Universidad de Antioquia. (b) Spectrographic representation of black‐striped sparrow song. In the figure are depicted the components of the song (introductory notes and trill)

We studied the Black‐striped sparrow (*Arremonops conirostris*, Figure 1a), a Neotropical songbird occurring in weedy fields and secondary growth across Central America and Northern South America. This species was ideal for our study for several reasons: (i) it is territorial; (ii) males advertise from the same trees throughout consecutive days, which simplifies territory mapping; (iii) it is abundant in our study area, with males often in close proximity; (iv) its song has a trilled component that is easily detectable and suitable for vocal performance analyses; and (v) it is frequently affected by avian malaria, with a prevalence of about 50% in our study area.

Avian malaria is a vector‐borne disease caused by intracellular protozoan parasites of the genus *Plasmodium*, *Haemoproteus*, and *Leucocytozoon* (Lapointe et al., 2012) and transmitted by insects of the order Diptera that have a worldwide distribution. This disease has been detected in tropical countries in bird species of multiple orders and families (Gonzalez‐Quevedo et al., 2016; Pulgarín‐R et al., 2018).

### 2.2 Recording and sampling

We recorded and sampled 19 Black‐striped sparrows at four different sites in Antioquia, Colombia, during the breeding season May–June 2017. All sites were located in tropical dry forest, between 400 and 700 m.a.s.l., with average temperature of 25°C and 1000 mm precipitation per year. This is a highly seasonal deciduous forest, with a long dry season. The Black‐striped sparrow is a common species that inhabits shrubs in this habitat (Rivera‐Gutierrez et al., 2018). Males are highly territorial and very vocal (Rivera‐Gutierrez et al., 2018), making them easy to find and follow.

Each individual male´s song was recorded during one dawn chorus. A single dawn chorus has been found to be sufficient for identifying the individual repertoire in several species (Rivera‐Gutierrez et al., 2011; Thompson et al., 2020; Zsebők et al., 2017). Territories were located and on the next morning, our recordist (SL) positioned herself at the border of the territory before dawn. Recording started from the first vocalization and lasted for 40 minutes. Recordings were collected using a Marantz PDM 661 recorder and a Sennheiser ME67 unidirectional microphone in WAV format, sampling rate 44 KHz, 16 bit.

Recorded males were then captured using mist nets at each bird´s territory. Captured individuals were categorized as “Adult” or “Immature”—immature birds can be identified by their yellowish‐olive head, brownish‐olive underparts, yellow bill at the base, and yellow gape flange. Additionally, adult males hold territories in which they use the same area to display their song behavior, and no immature was found holding a territory or singing. Individuals were weighed, measured (tarsus length), sampled for blood (brachial vein), and marked with a unique combination of colored leg bands to allow visual identification in the field. Blood samples were stored in lysis buffer. DNA was extracted using a standard sodium chloride extraction method (Gonzalez‐Quevedo et al., 2016), and DNA was checked for purity and concentration using a Nanodrop.

### 2.3 Acoustic analysis

We visually inspected sonograms of all recorded males (*N* = 19) to identify song repertoires and determine quality of the recordings (signal‐to‐noise ratio). This was done using Avisoft (Avisoft SAS‐LAB Pro V. 5.2, Berlin, Germany), with the following spectrogram parameters: Hamming window, FFT Length 512, frame size 75%, overlap: 50%. After this, we selected recordings of 14 males that were of sufficient quality for further analysis. Males’ songs are comprised of introductory notes followed by trilled syllables composed by as many as three rapid, frequency‐modulated notes (Figure 1b). Individuals display a repertoire ranging between 3 and 26 different song types (mean = 12.1). Given this variability in repertoire size, we estimated repeatability of song rate within individuals by using the *rptR* package (Stoffel et al., 2017) in the statistical software R (R Core Team, 2020), using the bootstrap method with 1000 boots for Poisson data. Repeatability was 0.45 ± 0.1 SE (CI= [0.221, 0.635]), which, though lower than ideal, is safely within range of repeatability for behavioral data (Bell et al., 2009). We, therefore, considered average values of song performance for our analysis. We used a mean of 4 exemplars per song type, only considering the trill portion of the song, and used spectrograms (parameters as above) to calculate the mean trill rate (notes/second) and mean trill frequency bandwidth. Trills were selected in Avisoft using an automatic selection method with a −30 dB threshold relative to the peak amplitude of the trills. This threshold excluded background noise while capturing variation within the frequency characteristics of the song.

Acoustic traits can be divided in those limited by physical constraints, known as index signals, and those limited by individual investment, also known as handicap signals (Bradbury & Vehrencamp, 2011; Gil & Gahr, 2002). Song performance‐related traits are a common example of handicap signals that indicate individual quality. These characteristics are a group of qualitative acoustic features that are variable in time and are limited by individual energy budgets. On the other hand, it is unlikely that performance traits represent physical limitations, such as neuronal costs or developmental stress (Gil & Gahr, 2002). Therefore, individuals may modulate expression of song performance traits according to their skill or available energy (Podos et al., 2009).

For estimating vocal performance, we calculated an upper bound regression following Podos (Podos, 1997), where trill types are binned by trill rate in 1‐HZ increment and the maximum frequency bandwidth within each bin is chosen to create a subset which is then plotted as a function of trill rate. This linear regression represents the performance limit for the trade‐off between trill rate and frequency bandwidth (Podos, 1997, 2001). For our study, vocal performance was estimated as the vocal deviation from the performance limit for the study population, measured as the minimal orthogonal distance from each trill type by male to the upper‐bound regression line (Podos, 1997). Values were averaged per male. The vocal deviation is thus a measure of relative vocal performance, with lower deviations (points closer to the line) representing higher performance songs and *vice versa* (Ballentine et al., 2004).

### 2.4 Malaria diagnosis and qPCR

We confirmed the sex of the individuals by molecular sexing, following Griffiths et al. (1998). Molecular sexing has added value as a further check on DNA quality. We then evaluated the presence of *Plasmodium* spp., *Haemoproteus* spp., and *Leucocytozoon* spp. in all captured males using the nested PCR protocol of Hellgren et al. (2004), optimized for our laboratory (Gonzalez‐Quevedo et al., 2016). The evaluated samples were Sanger sequenced by Macrogen Inc., Korea, to determine which lineage was infecting our individuals. Sequences were only positive for *Plasmodium homopolare*.

We developed a TaqMan qPCR approach to quantify *P*. *homopolare* parasitemia in the recorded males (*N* = 14): Species‐specific primers were designed to target a specific segment of the cytochrome b gene, Left Primer (5´CCTTGGGGTCAAATGAGTTT 3´), Right Primer (5´CCCTAAAGGATTTGTGCTACC3´) and the parasite *cyt b* TaqMan probe (/56‐FAM/TCTTGTTTCATGGATCTGTGGTGGA/3BHQ_1/) was labeled with FAM as a reporter and 3BHQ1 as a quencher. This probe was designed to be highly specific to its target sequence. The host mitochondrial gene ND4 was amplified using primers ND4 and Leu (Arévalo et al., 1994).

Amplified parasite *cyt b* fragments were cloned in Pgem‐T Easy vector, following the recommendations of the manufacturer (Promega), and recombinant plasmids were purified using the QIAprep Spin Miniprep Kit (QIAGEN). We performed serial dilutions (1:1 to 1:10000 plasmid) to set calibration curves for quantification of *Plasmodium* cyt b copies present in each DNA sample. qPCR analyses were carried out in a CFX 96 Real Time PCR Detection System (Bio‐Rad) using the following PCR preparation: 10 µl of Master Mix Quantinova Probe PCR KIT (Top Taq), 0,5 µl of each designed primer, 0,8 µl of the probe, 2 µl of DNA, and 6,2 µl of purified water for a final reaction volume of 20 µl, with the following thermal profile: 3 min at 94°C, followed by 35 cycles of 94°C for 30 s, 55°C for 30 s, and 72°C for 30 s, finalized by 3 min at 72°C. Individual parasitemia was estimated as the number of copies of the parasite genome detected in 2 µl of total DNA.

### 2.5 Effect of individual quality on vocal performance

We performed general linear models to evaluate the effect on male´s vocal performance of individual quality measures: tarsus length, body mass, and parasitemia (qPCR) served as predictors, and vocal performance as response. All possible models were run using the MuMIn package (Bartoń, 2020) in R, and the best models were chosen following the AICc criterion. A similar procedure was performed using diagnostics (absence/presence) instead of parasitemia. Normality of residuals was checked using PP plots. Script and data for GLM are provided in Appendices S1 and S2 respectively.

## 3 RESULTS

### 3.1 Acoustic parameters

**FIGURE 2:**
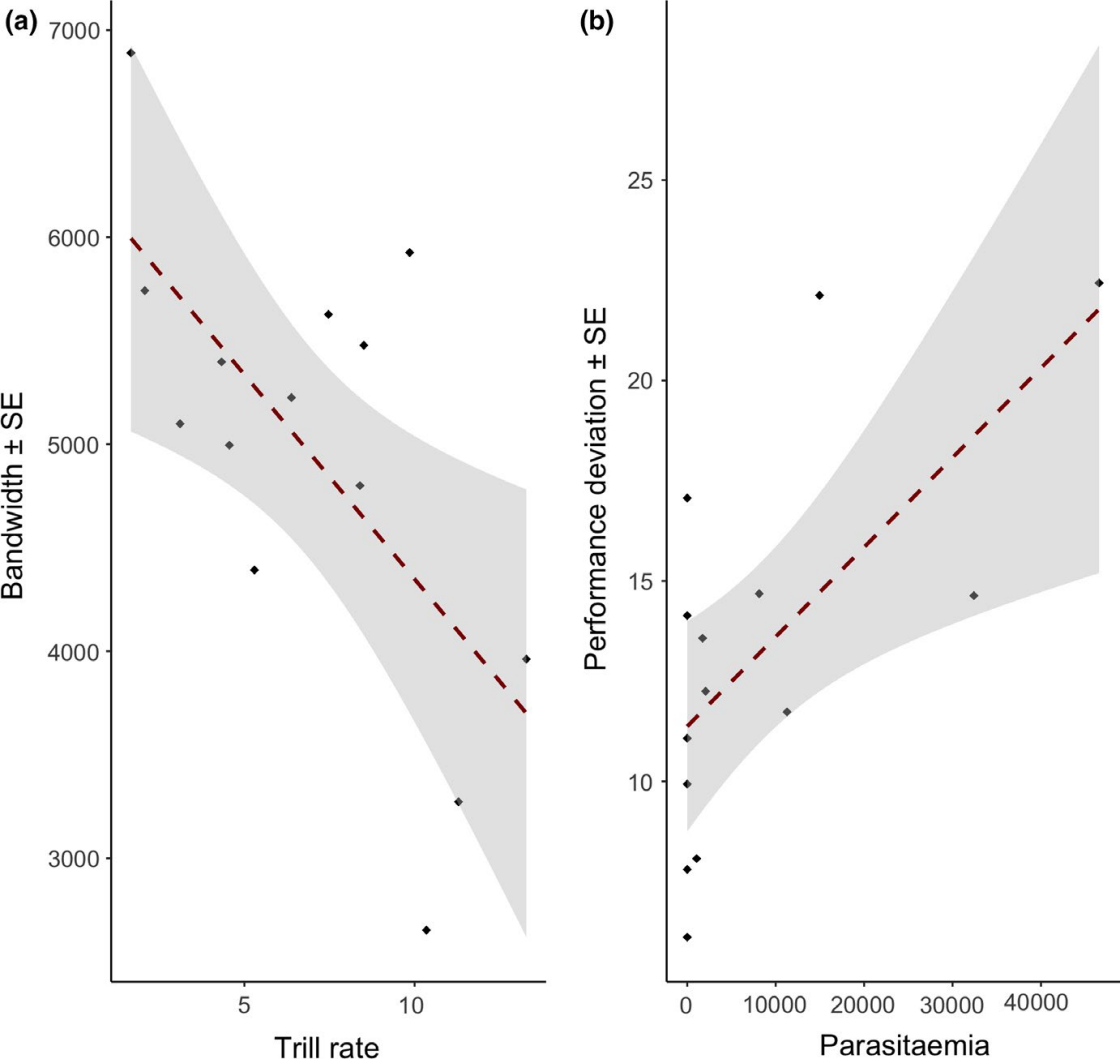
Performance analysis of black‐striped sparrow song. The panels illustrated the relationship between (a) bandwidth and trill rate; and (b) performance deviation and parasitemia levels

Frequency bandwidth was found to correlate negatively with trill rate for all songs (*p* < .05, *R* ^2^ = 0.4), resulting in a triangular distribution, as expected when frequency bandwidth is moderated by a trade‐off between the two measures (Podos, 1997) (Figure 2). Further analysis of within and between song variation in trill characteristics was beyond the scope of this study.

### 3.2 Malaria diagnosis and qPCR

All malaria‐positive males were infected by only one lineage, *Plasmodium homopolare* (ZOCAP11; Bensch et al., 2009). This is the first report of this *Plasmodium* strain infecting the Black‐striped sparrow, with a total prevalence of 44% (*n* = 50) in our surveyed community. In our subset of sampled males, the malaria prevalence was 57%, and there was within‐species individual variation in parasitemia (range: 0–46571 relative fluorescence units, Figure 2).

### 3.3 Effect of individual quality on vocal performance

We ran a total of 16 models with different combinations of predictors (8 including parasitemia, 8 including presence/absence). Models including diagnostic had lower performance than the models using parasitemia (Table 1). Binary infection status was not significant in any of the models. Parasitemia was present in four out of eight models, and the sum of weights was 0.8. Three models including parasitemia best explained variation of vocal performance (delta AICc lower than 2). We ran the most explicative models individually, and parasitemia was significant in two of them and had a nonsignificant trend in the third one (Table 2), having a negative relationship with vocal performance (Figure 2). Tarsus length and body mass were present independently in one model each, and they were not statistically significant. An average model included all factors and parasitemia was statistically significant (Table 2).

**TABLE 1:** Summary of all models of parasitemia, ordered by AIC

Model	Intercept	Body mass	Parasitemia	Tarsus length	df	logLik	AIC	Delta AIC	Weight
1	139.5	–	0.006919	–	3	−84.566	175.1	0	0.424
2	953.5	–	0.006327	−31.33	4	−84.133	176.3	1.14	0.24
3	−138.4	8.288	0.00636	–	4	−84.454	176.9	1.78	0.174
4	701.2	6.287	0.005933	−29.73	5	−84.066	178.1	3	0.094
5	−938.4	33.32	–	–	3	−87.534	181.1	5.94	0.022
6	503.7	27.38	–	−48	4	−86.839	181.7	6.55	0.016
7	1843	–	–	−63.72	3	−87.863	181.7	6.6	0.016
8	197.9	–	–	–	2	−88.969	181.9	6.81	0.014

**TABLE 2:** Summary of best models

Model	Term	Estimate	SE	*t*	*p*	*R* ^2^
1	Intercept	139.500	34.430	4.051	.00161**	0.42
	Parasitemia	0.007	0.002	3.242	.00706**	
2	Intercept	953.528	973.149	0.98	.3482	0.52
	Parasitemia	0.006	0.002	2.782	.0178*	
	Tarsus	−31.329	37.429	−0.837	.4204	
3	Intercept	−138.400	662.100	−0.209	.8382	0.53
	Parasitemia	0.006	0.003	2.466	.0313*	
	Body mass	8.288	19.720	0.42	.6823	
Average model	Intercept	33.820	40.140	0.843	.3995	
	Parasitemia	0.00020	0.00009	2.160	.0307*	
	Tarsus	−1.012	1.383	0.732	.4643	
	Body mass	0.115	0.458	0.251	.8015	

Note

Significance codes: “**” .01 “*” .05. Since we used deviation from vocal performance as response variable, a positive relationship between predictor and response indicates a negative relationship with vocal deviation and vice versa.

## 4 DISCUSSION

We evaluated the effect of malaria infection on a well‐known sexually selected character, song performance, in a common songbird. In our study, we used a novel technique for quantifying parasitic load, which may help to understand with greater precision how parasite infection can impact the evolution of sexually selected traits. As shown previously for many passerine species (Podos et al., 2009), we have found in the Black‐striped sparrow a trade‐off between trill rate and frequency bandwidth, shown in the triangular distribution and the negative slope of the regression line between these two song traits (Figure 2). As expected, when a trilled song is limited by a performance constraint, individuals producing songs at higher rates produced notes at lower bandwidths. This reflects the hypothesis that both features depend on movements of the vocal motor system (respiration, vocal tract, beak), with a trade‐off between the speed and breadth of such movements (Janicke et al., 2008; Podos et al., 2009).

Our main result indicates that individuals having the highest parasitemia displayed the highest vocal deviation from the mean vocal performance. This indicates that these individuals performed less challenging songs, thus revealing a negative correlation between a secondary sexual trait and parasitism. It has long been hypothesized that parasites have detrimental effects on their hosts, and infection has indeed been found to impact sexually selected traits (Hamilton & Zuk, 1982). The effect of parasitism on sexually selected characters has effects been found in traits such as tail length in newts (De Lisle & Rowe, 2015), wing spots in damselflies (Suhonen et al., 2018), plumage coloration in birds (Edler & Friedl, 2011; Lumpkin et al., 2014), throat color in lizards (Molnar et al., 2013), and calling rate and call duration in frogs (Madelaire et al., 2013; Pfennig & Tinsley, 2002).

Although birdsong has long been recognized as a sexually selected character (Searcy & Andersson, 1986), few studies have addressed the effects of blood parasites on its evolution.

In the case of ectoparasites, a few studies have shown effects on song expression: tick infestation resulted in reduced song consistency in male canaries (Müller et al., 2013), and hen flea infestation resulted in reduced song duration in male great tits (Bischoff et al., 2009). Concerning blood parasites, previous findings (measuring parasitemia using blood smears) have indicated that haemosporidian infection can modulate signaling behavior and directly affect song features. In sedge warblers (*Acrocephalus schoenobaenus*), males infected with parasitic blood protozoans had reduced repertoire sizes (Buchanan et al., 1999). Similarly, male canaries (*Serinus canaria*) infected with malaria had reduced vocal complexity compared with healthy males, due to a reduction of the volume of a song nucleus in the brain (Spencer et al., 2005). In male mountain White‐Crowned Sparrows, males parasitized with malaria showed reduced consistency and song output after playback (Gilman et al., 2007), and in Tawny Owls, blood parasite load correlated with diminished call frequencies and ranges (Redpath et al., 2000).

Our results are thus consistent with the hypothesized negative relationship between parasites and the expression of sexually selected characters (Hamilton & Zuk, 1982) and support and extend previous studies investigating the role of haemosporidians in binary presence/absence terms. Our study further implemented a well‐known technique for quantifying DNA (qPCR) as a measure of parasitic load, but that had not previously been used in the context of sexual selection. qPCR is more sensitive than traditional PCR, and even more so when compared to conventional microscopy (Imwong et al., 2014) that often missed cases of lower parasitemia (Koepfli et al., 2015; Tadesse et al., 2017). Furthermore, a recent study analyzing parasite presence and load has found contrasting results between these metrics, suggesting a trade‐off between prevalence and infection intensity (Roth et al., 2021). In our study, we aimed to evaluate a continuum of parasite load to determine its potential effect on a song trait. By using qPCR, we were able to accurately detect and quantify parasitemia, as well as detect infections in individuals that might otherwise have been categorized as uninfected.

Quantifying the level of parasitemia is relevant because greater parasite loads may impart greater costs on the host. Infection intensity could be related to host condition in a reciprocal manner, where a poor‐condition individual is not able to mount an appropriate immune response, resulting in high parasite proliferation; on the other hand, a high‐intensity infection could also cause host poor condition (Beldomenico & Begon, 2010). There are increased costs of parasitism associated with higher levels of parasitemia (Brunner et al., 2005; Hicks et al., 2018; Isaksson et al., 2013; Sheldon & Verhulst, 1996). These costs include the following: (i) loss of resources extracted by the parasite directly from the host; (ii) competition between the parasite and the host for resources; (iii) costs to the host for defense against parasites; and (iv) costs resulting from tissue injury either directly caused by the parasite or from the inflammatory and immune response to the parasite (Wobeser, 2008). Hence, a higher parasitemia will result in a higher cost of bearing or clearing parasites for the host.

Avian malaria produces inflammation that is coupled with increased oxidative stress, even at lower levels of parasitemia (Christe et al., 2012). Oxidative stress can have negative impacts on birdsong performance, because the brain is vulnerable to damage caused by reactive oxygen species (Casagrande et al., 2016). Moreover, oxidative stress induced by testosterone has been linked as a mechanistic basis for ensuring honesty in secondary sexual signals (Baldo et al., 2015). Thus, malaria‐infected males could potentially be facing a twofold cost of oxidative stress when singing, which could in turn result in reduced vocal performance. Malaria infection also decreases hemoglobin concentration (Krams et al., 2013) and affect hosts’ ability to transport oxygen in their blood (Gilman et al., 2007), which could negatively affect a demanding aerobic activity such as singing (Gilman et al., 2007). Such a link between performance characters and health state has been found in several bird species (Buchanan et al., 1999; Duffy & Ball, 2002; Gilman et al., 2007; Laiolo et al., 2007; Redpath et al., 2000).

Another factor that could impact vocal performance is age. In the Swamp sparrow (*Melospiza georgiana*), age has been shown to correlate negatively with vocal deviation (Ballentine, 2009; DuBois et al., 2011). Because our study was conducted in a wild population that is not monitored seasonally, and because our species cannot be aged by plumage beyond c. four months, it was not possible for us to determine a particular age of the individuals that were evaluated. Thus, though we can be sure from plumage and behavior that all of our assayed individuals were adult males, we are not in a position to test among‐adult effects of age.

If parasite burden limits the expression of sexually selected traits, receivers should prefer vigorous displays because they are associated with low/no parasitism. Receivers should thus adjust their behavior according to the information they perceive about the parasite burden of the singer (Garamszegi, 2005). The choosy sex might choose differentially in a parasite‐mediated system because less parasitized mates that are not fighting off a disease may be able to make a higher investment into reproductive behavior (Møller, 1990) or because they can transmit parasite resistance genes to the offspring (Hamilton & Zuk, 1982). In the first scenario, there would be direct benefits to the female of being choosy because the mate would be a better helper in the rearing of the young. In the second, the fitness benefits to the receiver would be indirect, because it would be the descendants who gain the direct benefits of the “good” genes.

In our study system, the choosy sex is the female. Although we did not measure whether females of this species are indeed receptive to vocal performance as a measure of mate quality recognition or health status, studies in other species have shown that this trait can be influenced by parasites and linked to male reproductive success (Ballentine et al., 2004; Janicke et al., 2008; Kleindorfer et al., 2019). As proposed by Hamilton and Zuk (1982), if females can perceive variations in the expression of the trait, they might benefit from choosing males with lower parasitic load or superior immune systems. Future work could look at parentage patterns in broods, while also ascertaining male parasitemia.

The expression of behavioral traits can be restricted by performance constraints, which implies the existence of limits to a signaler's ability to execute such behavior (Searcy & Nowicki, 2005). Displays associated with sexual selection should be subject to evolutionary constraints to ensure honesty, as to indicate that maximum performance should provide a honest indication of quality to the receiver (Janicke et al., 2008). Variation in display performance could hold functional value (Podos et al., 2016), and in the field of bioacoustics, the vocal performance metric has been adopted by an increasing number of behavioral ecologists, parting from traditional measures of quality such as repertoire size, a feature recently challenged by studies that have found that it might not be as prevalent or important for reproductive success as previously proposed (Sakata & Vehrencamp, 2012). Measurements of vocal performance can provide a reliable indication of condition because they involve more complex motor patterns (Ballentine et al., 2004; Podos, 1997, 2001). In addition, a quantitative measurement of parasitic load provides greater experimental power. Therefore, studies using song performance traits and objective measurements of parasitemia offer a helpful perspective for understanding evolution of characters via sexual selection (Sakata & Vehrencamp, 2012).

## CONFLICT OF INTEREST

All authors certify that they have no affiliations with or involvement in any organization or entity with any financial interest or nonfinancial interest in the subject matter or materials discussed in this manuscript.

## AUTHOR CONTRIBUTIONS

**Salome Lopez‐Serna:** Conceptualization (equal); Formal analysis (equal); Investigation (equal); Methodology (equal); Writing – original draft (lead). **Catalina González‐Quevedo:** Conceptualization; Investigation; Methodology; Writing – review & editing (equal). **Hector Fabio Rivera‐Gutierrez:** Conceptualization; Formal analysis; Funding acquisition (lead); Investigation; Methodology; Project administration (lead); Writing – original draft (equal); Writing – review & editing (equal).

## Supporting information

Appendix S1

Appendix S2

## Data Availability

Data and code for statistical analysis are publicly available at Dryad. https://doi.org/10.5061/dryad.sbcc2fr6f
